# An Italian Guidance Model for the Management of Suspected or Confirmed COVID-19 Patients in the Primary Care Setting

**DOI:** 10.3389/fpubh.2020.572042

**Published:** 2020-11-24

**Authors:** Noemi Lopes, Federica Vernuccio, Claudio Costantino, Claudia Imburgia, Cesare Gregoretti, Salvatore Salomone, Filippo Drago, Giuliano Lo Bianco

**Affiliations:** ^1^Provincial Health Authority of Palermo, Palermo, Italy; ^2^Centro Neurolesi Bonino Pulejo Istituto di Ricovero e Cura a Carattere Scientifico - Scientific Institutes of Hospitalization and Care (IRCCS), Messina, Italy; ^3^Department of Science for Health Promotion and Mother to Child Care, University of Palermo, Palermo, Italy; ^4^Section of Radiology - Department of Biomedicine, Neurosciences and Advanced Diagnostics, University of Palermo, Palermo, Italy; ^5^Department of Health Promotion Sciences, Maternal and Infant Care, Internal Medicine and Excellence Specialties “G. D'Alessandro”, University of Palermo, Palermo, Italy; ^6^Infectious Disease Unit, National Relevance Hospital Trust, Azienda Ospedaliera di Rilievo Nazionale e di Alta Specializzazione Civico Di Cristina e Benfratelli, Palermo, Italy; ^7^Department of Surgical, Oncological, and Stomatological Sciences, University of Palermo, Palermo, Italy; ^8^Anesthesiology and Pain Department, Fondazione Istituto G. Giglio, Cefalù, Italy; ^9^Department of Biomedical and Biotechnological Sciences, University of Catania, Catania, Italy

**Keywords:** COVID-19, primary health care, general practitioners, severe acute respiratory syndrome, severe acute respiratory syndrome coronavirus 2, SARS virus, coronavirus infections

## Abstract

An outbreak of Severe Acute Respiratory Syndrome Coronavirus 2 started in China's Hubei province at the end of 2019 has rapidly become a pandemic. In Italy, a great number of patients was managed in primary care setting and the role of general practitioners and physicians working in the first-aid emergency medical service has become of utmost importance to coordinate the network between the territory and hospitals during the pandemic. Aim of this manuscript is to provide a guidance model for the management of suspected, probable, or confirmed cases of SARS-CoV-2 infection in the primary care setting, from diagnosis to treatment, applying also the recommendations of the Italian Society of General Medicine. Moreover, this multidisciplinary contribution would analyze and synthetize the preventive measures to limit the spread of SARS-CoV-2 infection in the general population as well as the perspective for vaccines.

## Introduction

An outbreak of unexplained low respiratory infections cases detected in Wuhan (the largest metropolitan area in China's Hubei province accounting for 11 million inhabitants), was first reported to the WHO Country Office in China, on December 31, 2019 (1). The etiology of this illness was attributed to a novel virus belonging to the coronavirus (CoV) family, named Severe Acute Respiratory Syndrome Coronavirus 2 (SARS-CoV-2), originated from a reservoir of bats and unknown intermediate hosts (2–4). Previous outbreaks of coronaviruses (CoVs) such as the severe acute respiratory syndrome (SARS-CoV) in 2002 and the Middle East respiratory syndrome (MERS-CoV) in 2012 have been recorded previously and properly contained by Public Health Authorities in the past (5).

SARS-CoV-2 is a member of subgenus Sarbecovirus (previously lineage b) in the family Coronaviridae, genus Betacoronavirus, with high (87.6–87.8%) genome sequence identities to SARSr-Rp-BatCoV-ZXC21/ZC45, detected in Rhinolophus pusillus bats from Zhoushan, China, during 2015 (4). The outbreak of SARS-CoV-2 was declared a Public Health Emergency of International Concern on 30th of January 2020, when counted over 7,800 confirmed cases in China and 82 cases outside China (6). At that time, in Italy, only three imported cases (a couple of Chinese tourists and a University researcher back from the Hubei Province) were laboratory-confirmed and hospitalized at the “Lazzaro Spallanzani” Hospital in Rome (7). On 11 February 2020, WHO specified a new name for the disease caused by SARS-CoV-2: COVID-19 (2). Due to the characteristics of the novel Coronavirus (contagious for a long time, also in asymptomatic hosts) and to the extraordinary spread capacity (estimated R0 from 2.5 to 5.5), SARS-CoV-2 has reached the necessary epidemiological criteria to be declared a pandemic by the WHO on 11th March 2020, when more than 400,000 people in at least 150 countries had already been infected (8).

In Italy, the epidemic outbreak was in northern Italy, and this is probably due to the factors that may have influenced the sharp increase in the outbreak, including higher pollution and the presence of airports with international connections (9, 10). The rate of patients in home isolation without symptoms or with mild symptoms in Italy has progressively increased from 35% on March 11th when over 10,000 people were infected by SARS-CoV-2, to >95% on August 31st with more 268,000 infected people (11). Therefore, the role of the general practitioner in the context of the SARS-CoV-2 outbreak is crucial both for early detection of suspected patients and for the high rate of infected patients that are in home isolation and can be safely managed in the primary care setting. The aim of this manuscript is to provide a model for the management of suspected, probable, or confirmed cases of SARS-CoV-2 in the primary care setting, ranging from diagnosis to treatment.

Moreover, this multidisciplinary contribution analyze and synthetize the evidence based preventive measures that could limit the spread of SARS-CoV-2 infection in the general population as well as the perspective for vaccines.

## Covid-19: Infection, Transmission And Definition of Cases

SARS-CoV-2 is an RNA betacoronavirus and belongs to the coronaviridae family, which is considered an important human and animal pathogen. SARS-CoV-2 presents genetic regions similar to the SARS-Cov_1 and uses angiotensin-converting enzyme 2 as a cellular input receptor (12).

Compared to SARS-CoV-1 that replicates mainly in the lower respiratory tract, SARS-CoV-2 is highly present in the upper respiratory tract—even in asymptomatic patients—and this explains the greater transmission and spread caused by SARS-CoV-2 (13).

SARS-CoV-2 is transmitted via droplets and fomites during close unprotected contact, especially in enclosed places (14). Airborne transmission has not been reported for SARS-CoV-2 and it is not considered a way of contagion (15), although certain aerosol-generating procedures (i.e., orotracheal intubation) conducted in healthcare facilities could represent a way of airborne transmission (16).

However, patients undergoing procedures as noninvasive ventilation at home may be source of airborne transmission (17).

Fecal shedding has been demonstrated by some patients, but the fecal-oral route does not seem to be a possible way of SARS-CoV-2 transmission (14). The incubation period is approximately 14 days with most patients manifesting infection 4–5 days after exposure (18, 19).

Covid-19 cases are distinguished by WHO into suspected, probable and confirmed cases (20) as follows:

- Suspected cases:
  ° a patient with an acute respiratory tract infection presenting one of the following signs: cough, fever, shortness of breath AND no other etiology that fully explains the clinical presentation AND a history of travel or residence in a country/area reporting local or community transmission during the 14 days prior to symptom onset; OR
  ° a patient with any acute respiratory illness AND a close contact with a confirmed or probable COVID-19 case in the last 14 days prior to the onset of symptoms; OR
  ° a patient with severe acute respiratory infection with fever and at least one sign/symptom of respiratory disease (e.g., cough, fever, shortness breath) AND requiring hospitalization AND no other etiology that fully explains the clinical presentation.
- Probable Case: a suspected case testing for SARS-CoV-2 with inconclusive or positive results for a pan-coronavirus assay.
- Confirmed Cases: a case with laboratory confirmation of SARS-CoV-2 infection, irrespective of clinical signs and symptoms.
  Two main reason could be associated with a negative swab followed by a positive one after some days or weeks. The first one is associated with the low sensitivity of oro- and naso- pharyngeal swabs with possible false negative results. The second one could be related with the timing of the swab execution. SARS-CoV-2 demonstrate a time range of incubation that varied, 2–14 days but in some cases a positive swab was observed also 20 or 30 days after the suspect contact with a COVID-19 patient (21).

## Clinical Syndrome and Approach to Suspected or Confirmed COVID-19 Patients in the Primary Care Setting

The majority of people infected with SARS-CoV-2 (from 50 to 75%) are asymptomatic (22, 23), but the exact frequency of asymptomatic patients is difficult to establish yet. Asymptomatic confirmed cases may have lung abnormalities on chest CT and could represent “a formidable source” of contagion if adequate physical distancing measures lack (24–26). Interestingly, in a cohort of 58 asymptomatic patients admitted to hospital with an history of exposure to SARS-CoV-2, no symptoms and no alteration in laboratory findings, lung CT showed ground-glass opacity in about 95% of cases, and after short-term follow-up, 28% of them presented symptoms (27).

Symptomatic infection can be associated with different clinical illness (mild to critical form) (27). Most patients presenting to the general practitioner generally complain fever (body temperature > 37.5 in 89%), dry cough, shortness of breath (68%), anorexia (40%), productive cough (34%), myalgias (15%) or other suspected symptoms for COVID-19, such as olfactory and taste disorders (28–30). However, there is a major overall of symptoms of COVID-19 with other respiratory infections (e.g., flu from influenza virus) which makes it difficult to distinguish SARS-CoV-2 infection from them at an early stage. Another challenge for the general practitioner is the identification of cases at high risk of progression that may require hospitalization, considering that these patients are usually monitored through phone calls. Therefore, the general practitioner and physicians working in the first-aid emergency medical service coordinate the network between the territory and hospitals. For the abovementioned reasons, the Italian Society of General Medicine & Primary Care (S.I.M.G.), has published a guidance document (version 1.5 updated on April 29th, 2020) intended for general practitioners for the management of patients with suspected, probable or confirmed diagnosis of SARS-CoV-2 infection in the primary care setting (30). Herein, we illustrate the “ISVaMPIT” (i.e., Identify, Signal/report, eValuate, Monitor, Plan, Initiate Therapy) approach to these patients according to the guidance document, updated with information from current literature (30).

Patients with symptoms suspicious for COVID-19 should call their general practitioner and avoid going to his office, to first-aid emergency medical service, or the emergency department of hospitals. The general practitioner collects information regarding symptoms and epidemiological links with COVID-19 patients through phone calls and identifies suspected cases of COVID-19 (i.e., Identify of ISVaMPIT approach). Suspected cases of COVID-19 need to be reported to the local public health and hygiene service (i.e., Signal/report of ISVaMPIT approach) and appropriate precautions must be adopted for these suspected cases. Specifically, if the patient is asymptomatic the general practitioner should arrange fiduciary home isolation for him and his family for 14 days, and this quarantine can only be stopped in the presence of negative swab. In case of symptomatic patients the general practitioner will perform a telephone triage through a standardized form that allows to evaluate the severity of the symptoms and the presence of important comorbidities and risk factors and to decide whether to proceed with fiduciary home isolation, eventually request a swab and contact the special care continuity units (USCA), or to send the patient to the hospital for hospitalization by contacting the emergency/urgency service (30). If the symptomatic patient will not be hospitalized or in case of confirmed cases of COVID-19 not requiring hospitalization, the general practitioner should arrange fiduciary home isolation for him and his family for 14 days, and this quarantine can only be stopped in the presence of two negative swabs and clinical remission.

For the outpatient management of the COVID-19 or suspected COVID-19 patient by the general practitioner, personal protective equipment must be available in full (including the full suit or disposable gown, visor and, footwear as well as FFP2/FFP3 masks) (30, 31). If the personal protective equipment is not available, the general practitioner should not carry out the physical examination in his office or at patient home, but clinically evaluate and monitor the patient through telephone calls and/or, if possible, through video consultation (i.e., eValuate and Monitor of ISVaMPIT approach) (30).

Monitoring of clinical conditions includes daily monitoring of temperature, blood pressure (values ≤100 or ≥ 200 mmHg systolic indicate the severity of the disease), blood oxygen saturation, patient's state of consciousness, heart rate, and respiratory rate. Useful and practical tools for patient telephone monitoring of suspected or confirmed COVID-19 patients and prognostic evaluation include:

- the Mews score scale (Modified Early Warning Score), that gives a score based on the evaluation of five parameters (heart rate, body temperature, respiratory rate, alteration of consciousness, blood pressure) establishing the stability of the patient clinical condition (32);
- the “walking test,” which is performed by inviting the patient to walk for about 5 min with a finger pulse oximeter and then evaluating saturation at intervals of about 1 min; this test can reveal occult alterations in blood oxygen saturation in patients who do not experience desaturation at rest;
- CURB-65 score, which is an acronym for each of the risk factors measured (i.e., confusion of new-onset, blood urea nitrogen, respiratory rate, blood pressure, and age > 65 years old) and estimates mortality of community-acquired pneumonia (33, 34).
- Single breath counting (SBC) is an alternative test to assess pulmonary function. SBC is measured by asking patients to take a deep breath and count as far as possible in their normal speaking voice without taking another breath (35, 36).

In addition to monitoring the clinical status, the general practitioner will verify the adherence to preventive measures, the psychological status as well as the basic social and welfare patient conditions during these phone calls or video consultations.

For the outpatient management of suspected or confirmed COVID-19 patients who cannot be handled over the phone by the general practitioner or sent to the hospital, general practitioners can require the involvement of Special Units for Continuous Healthcare (USCA) or may send the patient to the special tents of the emergency departments of the hospital (i.e., Plan and Monitor of ISVaMPIT approach and as suggested by ministerial guidelines). USCAs have been set up in Italy to support primary care assistance in the setting of COVID-19 (37). Once activated by the general practitioner, the USCA team—using the suitable personal protective equipment—goes to patients' homes to perform a physical examination, assessment of blood oxygen saturation, eventual swab if needed, medical drug prescriptions, and, some first-level diagnostic tools (i.e., portable ultrasound, portable ECG) if available (38).The use of portable ultrasound in SARS-CoV-2 patients has emerged in the front lines of the SARS-CoV-2 epidemic in Italy (39). For instance, the predilection of lung pulmonary findings in subpleural regions and the availability of wireless ultrasound probes prompted to the possibility of using portable ultrasound as a potential triage and diagnostic tool in primary care setting after an adequate lung ultrasound training (37). Although there is limited experience at this time on US in SARS-CoV-2 patients, the main imaging findings in SARS-CoV-2 patients include thickening of the pleural line with pleural line irregularity, B lines in a variety of patterns including focal, multifocal, and confluent, consolidations in a variety of patterns including multifocal small, non-translobar, and translobar, A lines during the recovery phase, while pleural effusions were uncommon (38, 40–42).

Finally, in case of accidental or scheduled access to the general practitioner medical office of suspected cases of COVID-19, it is necessary to perform the sanitation of the environment according to the procedures indicated by the Italian National Institute of Health (Istituto Superiore di Sanità, ISS) (43).

## The Role of Swab in the Primary Care Setting

In Italy, there have been regional differences in the execution of the swabs. For instance, in the municipality of Vo' Euganeo in the Veneto region swab was performed to the entire local population based on the North Korean model, while in the remaining Italian regions, swab was performed only in some suspected cases of COVID-19 (44). On April 3, 2020, in Italy, the Ministry of Health indicated that swab should be reserved primarily to symptomatic/paucisymptomatic suspected cases of COVID-19 and symptomatic contacts of the suspected case (i.e., symptomatic contacts of the suspected case were defined as those who had contact with the suspected or confirmed case of COVID-19 in the 48 h before the onset of symptoms), health care professionals at high risk (45).

The general practitioner in the case of early/mild symptomatic suspected cases can request the swab by filling-in the telephone monitoring form and sending it to the territorial public authority that will take charge of the request and will perform the swab at the patient home (30). Based on the indication of the Italian Ministry of Health (45), some Italian regions have created “drive-through” COVID-19 checks in which people are referred to these checks by their local health authorities, queue in their cars to get tested and the swab is then performed from the relative safety of people's cars (46).

These testing strategies are generally preferred for patients who no longer have symptoms of COVID-19 and have to undergo the two consecutive negative laboratory PCR tests to be declared definitively “non-infecting,” in accordance with Italian guidelines of the Ministry of Health (47).

The CDC's criteria for patients to clear isolation, is based on two main strategies (48, 49):

1. Test-based strategy that represent the initial recommendation but still recognized (published on 12 January 2020), based on knowledge and experience with similar coronaviruses:
  - Resolution of fever without the use of fever-reducing medications AND
  - Improvement in respiratory symptoms (e.g., cough, shortness of breath), AND
  - Negative results of an FDA Emergency Use Authorized COVID-19 molecular assay for detection of SARS-CoV-2 RNA from at least two consecutive nasopharyngeal swab specimens collected ≥24 h apart (total of two negative specimens).
2. Non-test-based strategy, new WHO recommendation (published on 27 May 2020) that proposed:
  - For symptomatic patients: 10 days after symptom onset, plus at least three additional days without symptoms (including without fever and without respiratory symptoms)
  - For asymptomatic cases: 10 days after positive test for SARS-CoV-2.

These criteria can apply to all COVID-19 cases despite the type of specimen or disease severity and reflect recent findings that patients who no longer have symptoms sometimes stay positive for the COVID-19 virus (SARS-CoV-2) by molecular testing for many weeks. Regardless of the positive test result, these patients are unlikely to be infectious and transmit the virus. Although the risk of transmission after symptom resolution is likely to be minimal, it cannot be completely ruled out.

In symptomatic patients with a severe disease for prolonged periods of time, a laboratory-based approach measuring viral load and neutralizing antibody (or proven equivalent antibody) levels might be useful to decide for prolonged isolation.

At the moment, it is possible to use test-based strategy and in this case the initial recommendation of two negative PCR tests at least 24 h apart can be applied.

According to Italian guidelines, a Covid-19 patient is considered healed only after the resolution of symptoms and two negative consecutive tests for SARS-CoV-2 at 24-h intervals. Even if patients recover clinically before 7 days of onset of symptoms, it is still recommended to wait a 7-day interval between the first and last test. In asymptomatic patients, a negative SARS-CoV-2 RNA test at 14 days after the first test is needed (end of the quarantine period). For virus clearance it is defined as a negative viral RNA from body fluids of symptomatic and asymptomatic patients, accompanied by appearance of specific IgG (47). The discharge criteria do not allow the discontinuation of preventive measures, therefore, despite the negativity of the laboratory PCR test, the patient at home will still have to observe an isolation period for a total of 14 days. The optimum would be represented by the execution of a serological test that determines the presence of IgG. There is still no consensus on the need to repeat the serological test 30 days after the first determination.

Different approaches are provided in other countries. As an example, in France, a Covid-19 patient must remain in home isolation for at least 7 days since the onset of symptoms, and may leave home wearing a mask and respecting social distancing after these 7 days if he feels healthy; in case of persisting symptoms at the end of these 7-day quarantine, the patient must remain in home isolation and may leave home only 2 days after complete remission of symptoms, without the need for a new test for SARS-CoV-2 (50). In Germany, a positive test result means that the person concerned must self-isolate for 10 days; in most States of Germany, testing negative means home quarantine is no longer required, but in specific German States, a repeat test a number of days later may be necessary (51).

## Preventive Measures of SARS-CoV-2 Infection in the General Population

According to the WHO guidelines, some preventive measures should be carried out by general population to reduce the risk chances of being infected or spreading SARS-CoV-2 and the general practitioner should indicate them to his patients (52).

In particular, the following safety measures are considered essential in the prevention of the SARS-CoV-2 infection:

1. Regularly and thoroughly handwashing with soap and water or with an alcohol-based hand rub. If gloves are used, they should be change after usage or frequently. Hand washing remains the better way to prevent the virus spread;
2. Physical distancing (at least 1 meter or 3 feet distance between people) represents a second key point to prevent SARS-CoV-2 diffusion. For instance, when someone coughs, sneezes, or speaks they spray small liquid (called “droplets”) from their nose or mouth which could contain the virus, also if asymptomatic;
3. Avoid crowded places or events because it is difficult to avoid close contact with other people;
4. Avoid touching eyes, nose, and mouth with the hands. Avoid also to touch face if you are wearing gloves. Hands usually touch many surfaces and can pick-up viruses;
5. Follow correct respiratory hygiene such as covering mouth and nose with bent elbow or tissue when coughing or sneezing and after washing hands immediately;
6. Self and home isolation even with minor symptoms such as cough, headache, mild fever is necessary until a diagnosis of SARS-CoV-2. In case of necessity to leave the house wearing a mask is mandatory;
7. The use of personal protective equipment such as medical mask (without filters) is useful mainly in closed places such as markets, stores, shops, medical clinics, post offices, and bank offices.
8. It should be aware that coronaviruses can remain infectious on inanimate surfaces like metal, glass or plastic for up to 9 days. It is of utmost importance that surfaces and equipment like ventilators used for SARS-CoV-2 infected children should be carefully disinfected with about 62–71% ethanol, 0.5% hydrogen peroxide or 0.1% sodium hypochlorite within 1 min (53).

## Treatment for COVID-19 in the Primary Care and Hospital Setting

Regarding the therapeutic management of the patient to date, no therapies have been shown effective against SARS-CoV-2 and all treatments—although potentiality effective against COVID-19—need either appropriate drug development or clinical trial to be suitable for clinical use (54, 55). Therefore, the Italian Drug Agency (i.e., AIFA) has opened a section of its website where it constantly updates the therapies under investigation for Sars-CoV-2 infection (56).

There are few drugs available in the primary care setting.

In case of asymptomatic suspected or confirmed cases, no therapy is performed but only clinical monitoring. The potential role of probiotics in the treatment of SARS-CoV2 infection has been suggested by some studies as seem to be involved in reducing the expression of proinflammatory cytokines and in the restoration of microbial flora preventing secondary infections; however, products available for bacteriotherapy are not the same and have different potential effects (57, 58). In case of paucisymptomatic patients but with a negative swab or in the absence of swab, it is possible to start only a symptomatic therapy with paracetamol, which currently remains the first choice as antipyretic, cough sedatives and hydration and, if necessary, home oxygen therapy. Nevertheless, there are no clear indication for the amount of oxygenation to deliver at home. Patient develop silent hypoxia, namely hypoxia without dyspnea, in up to 18.7% of cases (19), and, therefore, ma refuse the use of oxygen.

If the patient is symptomatic and has a positive swab, it is important to discourage widespread use of antibiotics as their use may lead to higher bacterial resistance rates. Indeed, recently AIFA in the technical data sheet of azithromycin has discouraged its use if not in the presence of bacterial superinfection also because of its potential side effects (56).

The following therapies are instead mainly used in a hospital environment (59). First, both AIFA and FDA revoked the authorization for the use of hydroxychloroquine in COVID-19 patients because the potential risks, as i.e., prolongation of QTc, were greater than the possible beneficial effects (56, 60). It seems that therapy with corticosteroids is to be reserved for patients with more severe symptoms who require oxygen support, at a dose of 6 mg daily for 10 days or until discharge.

On June 2020, the European Medicines Agency first and then AIFA approved the use of Remdesivir—a novel nucleotide analog—as Emergency Support Instrument for adult and pediatric hospitalized COVID-19 patients with more severe clinical conditions (61, 62).

However, it is important to emphasize that Remdesivir is an investigational drug and that the authorization could be revoked on the basis of data that would demonstrate its non-safety. The suggested adult dose is 200 mg intravenously on day 1 followed by 100 mg daily for 5 days total (61, 62). The drug is not recommended for alanine aminotransferase values > 5 times the normal value and is not recommended in patients with glomerular filtrate < 30 ml/min unless there is a benefit greater than the risk of administering it (61, 62).

Another therapeutic strategy, initially tested in China, could be hyperimmune plasma containing specific antibodies against the virus, obtained by convalescent or hospitalized subjects, when there are no therapeutic alternatives. In Italy, the study TSUNAMI (TranSfUsion of coNvaleScent plAsma for the treatment of severe pneuMonIa due to SARS.CoV2) was authorized in May by the Ethics Committee of the Spallanzani Hospital in Rome aimed at evaluating the effectiveness of this experimental treatment which involved centers distributed in 12 regions on the Italian national territory (63).

Considering the risk of thromboembolic events, recently AIFA has also authorized a study on heparin (mainly with low molecular weight heparin, LMWH), in the light of recent evidence showing a better prognosis of patients treated with this drug, particularly in those with high D-dimer, as thrombotic processes in the vessels induced by the inflammatory cascade is described (56, 64, 65). However, long-term treatment with this drug could lead to important side effects, so the benefits of such a therapeutic protocol are still being evaluated. In patients with severely compromised clinical situations, home management should be focused on supportive and palliative therapy (66).

## Vaccination Against SARS-CoV-2: Where do we Stand?

The quick diffusion of COVID-19 across the world requires the development of effective therapeutic options against the disease caused by SARS-CoV-2. The absence of licensed vaccines or antiviral drugs forced clinicians to setup the treatment of COVID-19 disease mainly on their experience (67).

Usually, a vaccination exposes the body to an antigen that is unable to cause a disease, but that should elicit the immune response to limit or block the infection. Some of the technologies tested by researcher were previously used in vaccines licensed and offered to the general population. At the same time, different strategies that have not been used before in the production of vaccines are currently under trials (68).

At least six research groups have already begun phase 1 of the trial, injecting the first dose of the new vaccine developed into healthy volunteers, while other groups are experimenting the safety of the vaccine precursors on animals, as reported in a guide published on *Nature* in the month of April 2020 (69).

Several strategies are being tested to develop a vaccine against SARS-CoV-2 that could be resumed as follows (68–70):

- attenuated (live-attenuated) or inactivated virus vaccines: a type of vaccination that contain the SARS-CoV-2 in a weakened or inactivated form, that especially for live-attenuated vaccines requires several safety testing (71);
- viral-vector vaccines: engineering of other viruses (such as adenoviruses), replicating or not-replicating in a weakened form, to produce SARS-CoV-2 proteins, such as spike proteins (72);
- nucleic acid vaccines: these vaccines will encode the immunogenic proteins of SARS-CoV-2 (such as spike proteins) using the DNA or the RNA (73);
- protein-based vaccines: use of a fragment of proteins (protein subunits) or viral-like particles (VLP) of the SARS-CoV-2 to inject directly into the human body;

As of 31 July 2020, 7 months after the beginning of the SARS-CoV-2 pandemic, more than 165 vaccines are being developed worldwide and at least 19 vaccine candidates have, entered clinical trials, including phase 2 and 3 trials (74). Regarding the new vaccines against SARS-CoV-2, the major challenges for scientific community are the clinical safety and the effectiveness on a large scale, but also the construction of adequate platforms that could support vaccine production on a large scale (75).

In order implement vaccination plans and to guarantee the largest acceptance of new SARS-Cov-2 vaccines among at-risk categories such as subject with comorbidities, elderly or healthcare workers, previously studies conducted analyzed evidence-based strategies on how to reduce vaccine hesitancy (76–79).

To conclude, the SARS-CoV-2 outbreak is changing its face in Italy with an increasing number of patients managed in the primary care setting. The general practitioner and physicians working in the first-aid emergency medical service coordinate the network between the territory and hospitals. The role of the general practitioner in the context of the SARS-CoV-2 outbreak is crucial both for early detection of suspected patients and for the high rate of infected patients that are in home isolation and can be safely managed in the primary care setting. This article reviews the Italian model for the management of patients with suspected, probable, or confirmed diagnosis of SARS-CoV-2 infection in the primary care setting, providing insights from different specialties that may help in guiding patient management.

## Author Contributions

GL, NL, FV, CC, and CI: literature review and manuscript drafting. SS, FD, and CG: manuscript reviewing. All authors contributed to the article and approved the submitted version.

## Conflict of Interest

The authors declare that the research was conducted in the absence of any commercial or financial relationships that could be construed as a potential conflict of interest. The reviewer CL declared a shared affiliation with several of the authors SS, FD, and GL to the handling editor at time of review.
